# Supplementary material for: Human Sensory LTP Predicts Memory Performance and Is Modulated by the BDNF Val66Met Polymorphism
Source: Front Hum Neurosci. 2019 Feb 15;13:22. doi: 10.3389/fnhum.2019.00022 (PMC6384276; doi:10.3389/fnhum.2019.00022)
Supplement: Supplementary file 1 [file Table_1.pdf]

Spriggs, Thompson et al., 2019
Human Sensory LTP Predicts Memory Performance and is Modulated by the *BDNF* Val[66]Met Polymorphism

Supplementary material

Frequentist analyses

A linear regression analysis showed that *late LTP* was a reliable predictor of *Memory performance* ($R^2 = .196$, $R^2_{adj} = .165$, $RMSE = 7.28$). The correlation between *late LTP* and *Memory performance* was robust [$r = .44$, 95CI = (.08, .70), $p = .02$]. In contrast, *early LTP* was not a significant predictor of *Memory performance* ($R^2 = .00$, $R^2_{adj} = -.038$, $RMSE = 8.12$), and the correlation between the two variables was null ($r = .02$, $p = .92$).

An ANOVA on *LTP* with *BDNF genotype* as a fixed factor showed a significant effect of *BDNF genotype* [$F(2,25) = 14.61$, $p < .001$, $\eta^2 = .54$]. Pairwise comparisons showed a significant difference between Val/Val and Met/Met ($p < .001$, $d = 2.65$), between Val/Met and Val/Val ($p = .005$, $d = 1.41$), and between Val/Met and Met/Met ($p = .020$, $d = 1.11$). Note that the latter would not survive correction (FDR or Bonferroni), but all effects are presented uncorrected herein for comparative purpose with the Bayesian analyses.

An ANOVA on *early LTP* with *BDNF genotype* as a fixed factor showed a significant effect of *BDNF genotype* [$F(2,25) = 5.68$, $p = .009$, $\eta^2 = .31$]. Pairwise comparisons showed a significant difference between Val/Val and Met/Met ($p = .009$, $d = 1.30$), between Val/Met and Val/Val ($p = .007$, $d = 1.26$), but not between Val/Met and Met/Met ($p = .964$, $d = 0.02$).

An ANOVA on *Memory performance* with *BDNF genotype* as a fixed factor, showed a significant effect of *BDNF genotype* [$F(2,25) = 3.87$, $p = .03$, $\eta^2 = .24$]. Pairwise comparisons showed a significant difference between Val/Val and Met/Met ($p = .011$, $d = 1.33$), but not between Val/Met and Val/Val, or between Val/Met and Met/Met ($p = .122$, $d = 0.73$, and $p = .222$, $d = 0.57$, respectively). All effects are presented uncorrected herein for comparative purpose with the Bayesian analyses.
